# Shen-Cao granules formulated based on traditional Chinese medicine alleviates bone marrow suppression caused by platinum-based anticancer reagents

**DOI:** 10.1097/MD.0000000000006818

**Published:** 2017-05-12

**Authors:** Chunfeng Yu, Zhonghua Jiang, Aihua Hou, Yuejun Mu, Wei Liu, Song Tan

**Affiliations:** a Department of Oncology; b Department of Image, Yantai Hospital of Traditional Chinese Medicine, Yantai, China.

**Keywords:** chemotherapy, malignant tumor, thrombocytopenia, traditional Chinese medicine

## Abstract

**Background:**

The aim of this study was to evaluate effects of Shen-Cao granules for the prevention of thrombocytopenia caused by anticancer chemotherapy.

**Methods:**

In this prospective study, a total of 200 patients with various malignant tumors were enrolled and evenly divided into a Shen-Cao granule treatment (n = 100) and a control group (n = 100). After 2 cycles chemotherapy with any combination of platinum-based drugs (cisplatin, carboplatin, and nedaplatin), the blood platelet (PLT) counts, levels of the PLT production regulator thrombopoietin (TPO), PLT aggregation rates, and the PLT activation marker CD62P expressions were monitored for 2 weeks.

**Results:**

During 2 weeks of post-chemotherapy, the mean values of the minimum PLT count were 49.65 ± 7.35 × 10^9^/L in the treatment group and 31.56 ± 9.32 × 10^9^/L in the control group. The PLT count in the treatment group reached the lowest value 1.8 days later and recovered to a concentration ≥100 × 10^9^/L 3 days earlier than in the control group. The concentrations of the TPO were 71.43 ± 1.74 and 87.24 ± 0.92 ng/mL in the treatment group and 65.75 ± 1.39 and 67.75 ± 0.67 ng/mL in the control group at 7 and 14 days post-chemotherapy, respectively. The maximum PLT aggregation rate declined after chemotherapy in the treatment group from 58.14 ± 11.46% to 52.89 ± 10.52%, while it increased in the control group from 56.94 ± 10.55% to 61.75 ± 12.26%. Coordinately, the expression of CD62P in the treatment group decreased from 6.17 ± 0.59% to 4.89 ± 0.72%, while it increased from 6.09 ± 0.75% to 7.75 ± 0.67% in the control group.

**Conclusion:**

Our study demonstrated that Shen-Cao granule treatment alleviated thrombocytopenia after chemotherapy, and reduced tumor-induced PLT activation and aggregation.

## 1 Introduction

To date, one of the main means of treating malignant tumors is chemotherapy. However, the antitumor activity of these powerful agents also damages normal tissue to varying degrees, often leading to a series of adverse reactions in the body. Platinum-based antitumor agents are commonly used to treat solid tumor, which represents about 70% to 80% of all clinical chemotherapy for malignancy. They are widely used as first- or second-line treatment for cancer of the lung, cervix, ovary, head and neck, stomach, colon, and other solid tumors. However, the severity of the side effects limits the use of these drugs, particularly at high doses. Platinum-based antitumor drugs commonly target proliferating DNA, and their adverse effects include renal toxicity, gastrointestinal disorder, and bone marrow suppression. A variety of new platinum compounds with high efficacy and low toxicity have been developed and the adverse side effects, such as digestive tract disorder, renal toxicity, hair loss, and emergence of drug-resistance tumor cells within tumors, have been largely reduced, but bone marrow suppression remains unimproved.^[1,2]^ Bone marrow suppression refers to the decline in the activity of blood cell precursors in the bone marrow. Red and white blood cells (WBCs) as well as platelets in the peripheral blood are all derived from proliferating and differentiating hematopoietic stem cells in the bone marrow. As chemotherapeutic antitumor drugs target rapidly proliferating cells, hematopoietic stem cells are also severely affected, leading to a reduction in the number of red and WBCs and platelets in peripheral blood. In particular, a low blood platelet count, referred to as thrombocytopenia, causes bleeding into tissues, necessarily results in a reduction in the dose of chemotherapeutic drugs leading to failure of chemotherapy. Platelet transfusion is a standard treatment for severe thrombocytopenia, which can reduce the risk of fatal hemorrhages in important organs, and has an affirmative curative effect on transient thrombocytopenia caused by bone marrow inhibition. However, the clinical application of platelet transfusion is limited by various factors such as the supply and preservation of platelets, difficulties in procedure, a risk of graft versus host disease (GVHD) following an allogeneic tissue transplant, and infection risks from syphilis, HIV, and hepatitis associated with blood transfusion.^[3–6]^ In addition, 30% to 70% of all patients with chronic thrombocytopenia who received multiple platelet transfusions showed no response or suffered from platelet transfusion associated purpura.^[7]^

Traditional Chinese medicine (TCM) has been used as an adjuvant therapy to alleviate cancer symptoms at the terminal stages when western medicine cannot offer any other treatment options.^[8]^ However, recent studies indicated that TCM could play an important role in the entire course of cancer prevention and treatment.^[9]^ The usage and function of TCM varies depending on the stages of the cancer lesions.^[8,10]^ TCM creates favorable conditions for chemotherapy, reduces side effects such as bone marrow suppression, and improves the quality of patients’ life during chemotherapy as well as reducing medical expenses.^[10–12]^ Therefore, it is of great importance to develop and explore the effectiveness of TCM for the improvement of chemotherapy for cancer. Thrombopoietin (TPO) is a hematopoietic growth factor mainly expressed constitutively in the liver and kidney, which can extend the number of CD34+ and CD38- multipotential hemopoietic stem cells ^[13]^ and induces megakaryocytopoiesis and concomitant platelet formation. TPO binds to the myeloproliferative leukemia virus (c-Mpl) receptor on mature platelets and is subsequently destroyed, which acts as a regulatory negative feedback mechanism of TPO availability for megakaryocyte exposure to this hormone.^[14]^ Thrombocytopenia in patients with liver cirrhosis has been attributed to an imbalance of TPO expression in the liver and degradation by platelets sequestered in the congested spleen.^[15]^ TPO agonist treatment has been proposed for patients who develop thrombocytopenia as a result of cancer chemotherapy.^[16]^

At Yantai TCM Hospital, diseases such as hemopathy and bone marrow suppression have been categorized as consumptive disease or blood deficiency in Chinese medicine according to the clinical symptoms of patients. On the basis of TCM theory, clinicians at the hospital produce pharmaceuticals named Shen-Cao granules aimed at strengthening the liver and kidney, and reinforcing the spleen and Qi or energy. Subsequently, Shen-Cao granules have successfully been widely used to treat patients who developed bone marrow suppression after chemotherapy. As Shen-Cao granules affect liver and kidney functions and TPO (the major protein related to thrombocytopenia that is produced mainly in the liver and kidney), we speculated that Shen-Cao granule medications might improve thrombocytopenia by restoring TPO production in the liver and kidney in patients under chemotherapy.

The aim of the present study was to verify further the effectiveness of Shen-Cao granules for the prevention or control of thrombocytopenia of solid tumor patients, who were suffering from bone marrow inhibition due to chemotherapy. The effectiveness and safety of the Shen-Cao granules was evaluated for the development of improved clinical practice.

## 2 Methods

### 2.1 Recruitment of patients

The study was approved by the Ethics Board of Yantai Hospital of TCM and all participants signed consent forms. A total of 200 patients from our department volunteered to participate in this clinical study. All patients had been diagnosed with malignant tumors by imagology and pathology from April 2013 to June 2014 and had accepted at least 2 cycles of combination chemotherapy with 2 platinum-based drugs. The inclusion criteria included pathological or radiographic confirmation of malignant tumors; acceptance of 2 consecutive cycles of chemotherapy; performance status of Kamofsky (KPS) ≥60; ages from 18 to 80 years; life expectancy ≥3 months; normal function of bone marrow, heart, liver, kidney, and lung. The exclusion criteria included pregnancy; tuberculosis, severe infection, and mental disorders; uncontrolled blood system disorders; uncontrolled brain metastases.

### 2.2 Clinical methods

#### 2.2.1 Medications and platelet counts

All patients received 2 cycles of combination chemotherapy with any 2 types of platinum-based drugs, namely cisplatin (Hansoh Pharmaceutics, Jiangsu, China) carboplatin and nedaplatin (Qi-lu Pharmaceutics, Shandong, China). The patients were randomly divided into 2 groups of 100. The treatment group was given Shen-Cao granules (Table 1) and the control group a placebo granule (dextrin and caramel) (Beijing Kang Rentang Pharmaceutical Co. Ltd, Beijing, China). The appearance and packaging of the placebo was identical to that of the genuine drugs.

**Table 1:**
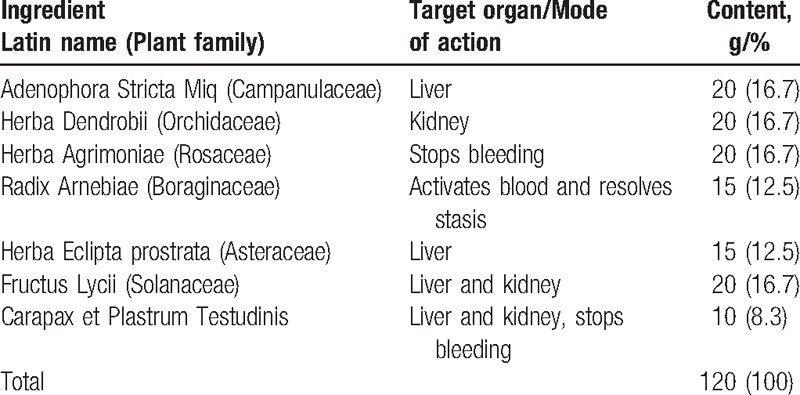
Ingredients of the Shen-Cao granules.

Patients in the treatment group were given Shen-Cao granules at the beginning of the first cycle of chemotherapy twice a day until the end of the 2nd cycle of chemotherapy. Patients in the control group took placebo granules in the same manner. We performed patients’ routine blood tests regularly during the chemotherapy. When the number of platelets declined to <75 × 10^9^/L, patients received only a supportive treatment to mitigate the symptoms, and their blood was tested at the next regular round. When the number declined to <50 × 10^9^/L, a routine blood test was carried out every day; when the number declined to <20 × 10^9^/L, patients received a platelet transfusion. If the WBC count was <4 × 10^9^/L, patients received a subcutaneous injection with recombinant human granulocyte colony stimulating factor (g-csf).

#### 2.2.2 Analyses of liver and kidney functions

Using a fully automatic biochemical analyzer (PUZS-300; Beijing Perlong New Technology Co. Ltd., Beijing, China), the functions of the liver and kidneys were measured with an alanine aminotransferase (GPT/ALT) assay kit (Beijing Shouyi Clinical Medicine Scientific Center, Beijing, China), an aspartate transaminase assay kit (Beijing Shouyi Clinical Medicine Scientific Center, Beijing, China), and a creatinine assay kit (YZB/JING 0029-2006; Beijing Shouyi Clinical Medicine Scientific Center, Beijing, China).

#### 2.2.3 Determination of peripheral blood thrombopoietin concentrations

The Sandwich ELISA method (TPO ELISA kit) (West Tang Biotechnology, Shanghai, China) was used to determine the concentration of peripheral blood TPO before as well as 7 and 14 days after chemotherapy. Briefly, blood from patients was centrifuged at 3000*g* for 10 minutes and 10 mL of serum was collected in a pyrogen/endotoxin-free tube. A standard containing TPO and serum samples was pipetted into each antigen-coated microplate well, followed by the addition of horseradish peroxidase (HRP) conjugate. After incubation and washing with PBS to remove the excess HRP conjugate, chromogenic detection was carried out with a TMB kit (West Tang Biotechnology, Shanghai, China). The values of optical density were measured at a wavelength of 450 × 2 nm.

#### 2.2.4 Blood platelet examinations

The peripheral blood PLT count was measured with an automatic hemocyte analyzer (BM860; Taian Tylenol Technology Trade Co., Ltd., Taian, China) 24 hours after the 2nd cycle of chemotherapy, and thereafter at 3, 7, 10, and 14 days, respectively. During post-chemotherapy, we recorded the minimum number of peripheral blood PLT counts, the length of time required to reach the minimum value, and the length of recovery time to a concentration of ≥100 × 10^9^/L.

*The PLT function was determined by an aggregation test of patients’ blood plasma using* turbidimetry with a platelet aggregation analyzer (LBY-NJ4; Beijing Precil Instrument Co. Ltd., Beijing, China). The degree of PLT activation was examined by measuring the expression of the PLT activation marker CD62P with a flow cytometry instrument (BD FACSCalibur; BD Biosciences, New York) before and 2 weeks after chemotherapy. The number of platelet transfusions each patient received was also recorded.

#### 2.2.5 Statistical analysis

SPSS software (SPSS for Windows, Version 13.0; SPSS Inc., Chicago, IL) was used for statistical analysis. Data are expressed as the mean ± standard deviation (SD). Statistical differences between the 2 groups were assessed using a *t* test, Chi-square test, or 2-way analysis of variance (ANOVA). *P* < .05 was considered to indicate statistically significant differences.

## 3 Results

### 3.1 Characteristics of patients

A total of 200 participants aged between 24 and 79 years (average age 54.63 ± 7.43) were randomly divided into the Shen-Cao granule treatment and the control group with 100 patients in each. The treatment group consisted of 61 males and 39 females with an average age of 53.2 ± 4.4 years. The control group consisted of 56 males and 44 females with an average age of 55.8 ± 5.1 years. The gender distributions between the 2 groups were not statistically different; however, the average ages were significantly different (*P* < .001) (Table 2). The sites of cancer and blood count values of pre-chemotherapeutic treatment, such as WBCs, neutrophil (NEUT), and hemoglobin (Hb) counts, showed no statistically significant differences between the 2 groups (*P* > .05).

**Table 2:**
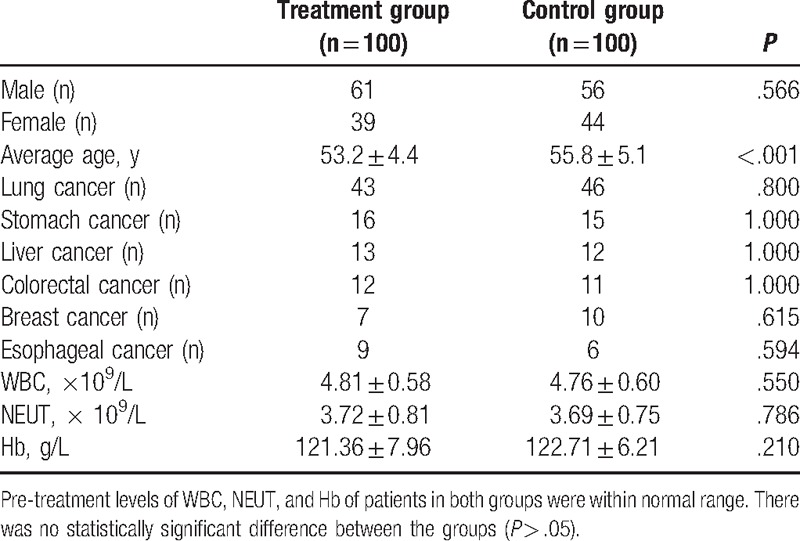
Preliminary patient clinical data (mean ± SD).

### 3.2 Evaluation of the effects of Shen-Cao granule treatment

#### 3.2.1 PLT count

**Figure 1:**
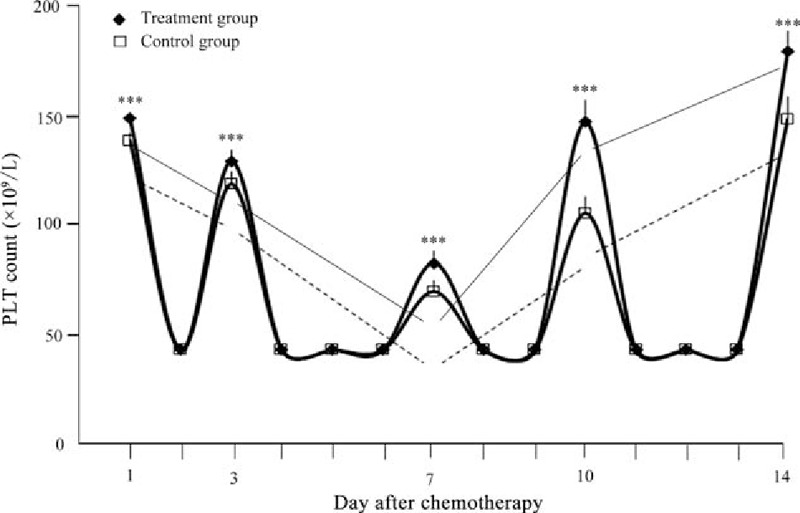
PLT concentrations in the peripheral blood of patients in the Shen-Cao granule treatment and the control group after indicated times of chemotherapy. ^∗^*P* < .001.

As one of the outcomes of bone marrow suppression caused by anticancer chemotherapy is a reduction in the blood PLT count, the effect of Shen-Cao granule treatment on PLT levels was evaluated. The PLT count in peripheral blood of patients declined after chemotherapy in both groups; however, they were significantly higher in the treatment group than in the control group at each time point (Fig. 1). The difference between the 2 groups was statistically significant (*P* < .001). The mean value of the minimum PLT count of the Shen-Cao granule treatment group (49.65 ± 7.35 × 10^9^/L) was higher than that of the control group (31.56 ± 9.32 × 10^9^/L) (Table 2). In the treatment group, the PLT count reached the lowest value 8.41 ± 1.54 days after termination of chemotherapy, while it took 6.62 ± 1.39 days in the control group. The mean length of the recovery time from the lowest values to a concentration ≥100 × 10^9^/L was shorter in the treatment group (5.76 ± 1.48 days) than in the control group (9.51 ± 2.13 days) (*P* <  .001) (Table 3). Four patients in the control group, whose PLT count declined to <20 × 10^9^/L and who were at a high risk of bleeding, received 12 units of platelet transfusion in total, while no patient in the treatment group required this therapy.

**Table 3:**
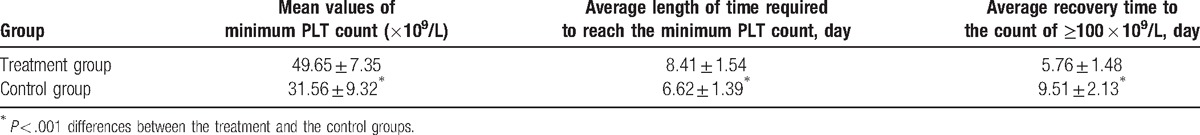
Comparison of the mean values of the minimum PLT count, the length of time for declining to the minimum value, and for recovery to a count ≥100 × 10^9^/L between the treatment and the control groups.

#### 3.2.2 TPO blood concentrations and PLT aggregation

**Figure 2:**
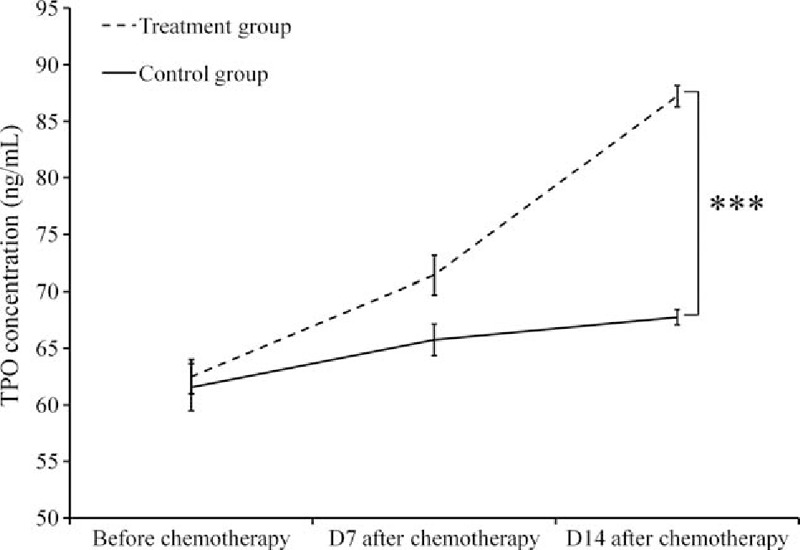
Two-way ANOVA test comparison of blood TPO concentrations before and day 7 and day 14 after chemotherapy between the Shen-Cao treatment and control groups. Note: ^∗^*P* < .001 between the treatment and the control groups.

The production of PLT is regulated by TPO, a hormone produced in the liver and kidneys.^[17]^ The concentration of blood TPO was measured before and after chemotherapy. The TPO levels 7 and 14 days after chemotherapy were significantly higher in the treatment group (71.43 ± 1.74 and 87.24 ± 0.92 ng/mL, respectively) than in the control group (65.75 ± 1.39 and 67.75 ± 0.67 ng/mL, respectively) (*P* < .001) (Fig. 2).

Platelet aggregation requires PLT activation and is an important process to stop bleeding at injury sites. However, it is involved in tumor growth, invasion, and metastasis.^[18]^ Therefore, Shen-Cao granules were assessed for their effects on PLT aggregation and activation before and after chemotherapy. The maximum PLT aggregation rate stayed within the normal range of 41% to 80.5% in both groups. However, it declined in the treatment group from 58.14 ± 11.46% to 52.89 ± 10.52%; in contrast, it increased in the control group from 56.94 ± 10.55% to 61.75 ± 12.26% after chemotherapy.

#### 3.2.3 PLT activation

The PLT activation was assessed before and after chemotherapy by measuring the cell surface expression of the PLT activation marker CD62P using flow cytometry. In the treatment group, CD62P decreased from 6.17 ± 0.59% to 4.89 ± 0.72% after chemotherapy; in contrast, it increased from 6.09 ± 0.75% to 7.75 ± 0.67% in the control group. Consequently, it was significantly lower in the treatment group than in the control group (*P* < .001) and coincided with PLT aggregation rates (Table 4).

**Table 4:**
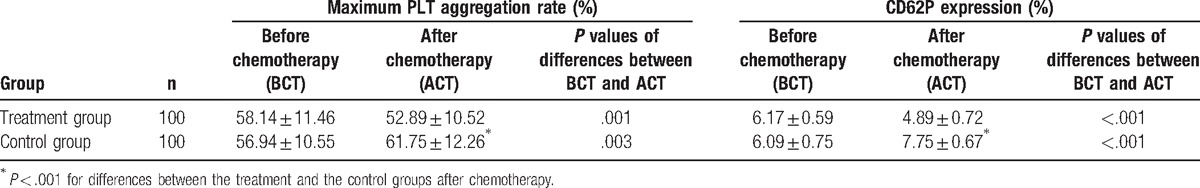
The maximum PLT aggregation rate and CD62P expression before and after chemotherapy.

#### 3.2.4 Liver and kidney functions

Compared with the control group, the ALT, aspartate aminotransferase, and creatinine serum concentrations were significant lower on day 14 after chemotherapy (*P* < .05) (Table 5).

**Table 5:**
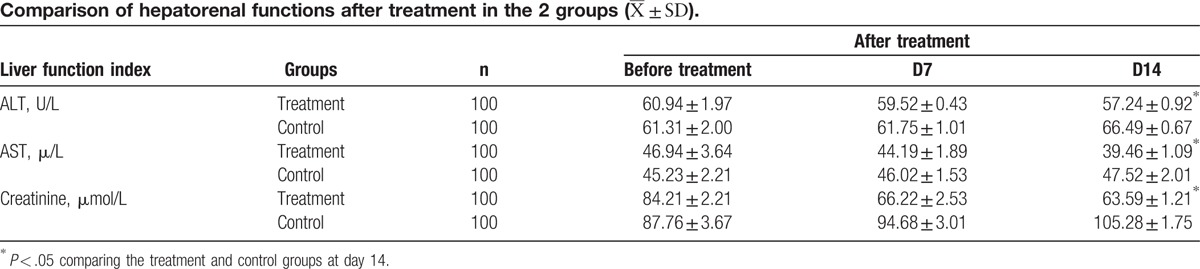


## 4 Discussion

Anticancer chemotherapy usually targets rapidly dividing cells in order to destroy cancer cells but inevitably damages normal cells that are rapidly dividing. Therefore, one of the serious side effects of cancer chemotherapy is the occurrence of myelosuppressive effects.^[16,19]^ In this study, we evaluated Shen-Cao granules, which were formulated on the basis of the theory of TCM, with respect to effects on bone marrow inhibition induced by anticancer chemotherapy. The most severe inhibition of bone marrow hematopoietic cells usually occurs 1 to 3 weeks after chemotherapy has been initiated.^[20]^ During 2 weeks of post-chemotherapy, which used a combination of platinum-based anticancer drugs, we measured the PLT count of patients and observed that the PLT count declined within 1 week. However, the counts were significantly higher at each time point than in patients who took placebo. The mean value of the minimum PLT counts was significantly higher in the Shen-Cao granule treatment group than in the control placebo group. Moreover, the PLT count in the treatment group reached the lowest value approximately 1.8 days later, and recovered to concentrations ≥100 × 10^9^/L about 3 days earlier than in the control group. In contrast to the control group, none of the patients in the treatment group required a transfusion because of extremely low PLT counts and the concomitant associated high risk of bleeding. These findings suggest that Shen-Cao granules can protect the bone marrow function from the adverse effects of anticancer reagents and promote platelet proliferation, thus alleviating thrombocytopenia. Thus, Shen-Cao granules might help patients to complete a planned cycle of regular anticancer chemotherapy without platelet-related complications.^[16]^

TPO is a regulatory factor produced by the liver and kidney. It binds to a specific receptor c-Mpl and stimulates the proliferation, differentiation, and maturation of the megakaryocyte, which is responsible for the production of platelet.^[17]^ It has also been reported that TPO is constitutively expressed in a variety of organs throughout the body, and that the blood TPO level is regulated by the total counts of both megakaryocytes and platelets as well as the total count of c-Mpl in the circulation.^[21]^ The relationship of the PLT count to the plasma TPO level was inversely correlated. TPO bound to the surface of PLT is destroyed leading to a reduction in plasma TPO levels. Therefore, a low PLT level results in a higher level of TPO leading to an increased chance of TPO to stimulate proliferation, differentiation, and maturation of megakaryocytes, which in turn increases the production of PLT.^[17]^ We measured the levels of peripheral blood TPO before as well as 7 and 14 days after chemotherapy, and found that in the Shen-Cao granule treatment group, the TPO level showed a dramatic increase and a significantly higher level during post-chemotherapy than the control group. If the effect of Shen-Cao granules was limited to the protection of bone marrow function, the plasma TPO level in the treatment group would not be higher than in the control group because of negative feedback between PLT and TPO. This result leads to an alternative hypothesis, namely that Shen-Cao granules stimulates TPO production and increases its plasma level. TPO is primarily produced and secreted into the blood by liver cells, bone marrow stromal cells, renal proximal and distal convoluted tubule cells, and the spleen.^[22]^ In our study, we found that Shen-Cao granules significantly improved liver and kidney functions (Table 5), which in turn might be the reason for enhanced TPO expression and resulting platelet activation. This possibility coincides with the theory of TCM, which emphasizes the restoration of the harmonious interaction of internal organs for the treatment of disease.

Platelet aggregation is an important physiological process in helping to form a hemostatic plug (thrombosis) at sites of vascular injury. However, tumor cells have a series of pathways and mechanisms to stimulate the release of microparticles from platelets that promotes platelet aggregation and also to stimulate the release of aggregates into the bloodstream.^[18]^ This tumor-induced platelet aggregation (TCIPA) enables the tumor cells to escape from the body's immune system by covering the cell surface with a layer of platelets, promotes tumor cell growth, invasion, angiogenesis and the formation of metastatic foci by facilitating adherence to vascular endothelial cells, and the release of tumor growth factors.^[19]^ Large aggregates of platelets produced by tumor cells readily lead to microvasculature embolism and cause serious consequences such as pulmonary embolism.^[23,24]^ Therefore, reducing platelet aggregation is one of the most important aspects to consider during tumor treatment. Our study has demonstrated that the maximum platelet aggregation rate after chemotherapy in the Shen-Cao granule treatment group was significantly lower than that in the control group, despite the fact that the blood PLT count of the former was significantly higher than in the latter. The results suggest that Shen-Cao granules influence the process of platelet aggregation, and could be used as a platelet aggregation inhibitor. We also measured the expression of the CD62P molecule on the membrane surface of platelet to investigate platelet activation status. In our study, the CD62P expression levels appeared to be higher (4.89 ± 0.72–7.75 ± 0.67%) than the normal range (0–2.8%) in both groups, before and after chemotherapy. This may be due to pre-existing platelet activation in patients with malignant tumors. However, after chemotherapy, the CD62P levels in the Shen-Cao granule treatment group dropped but in contrast increased in the control group, resulting in a significant difference between these 2 groups. As platelets have to be activated before aggregation, this result correlates well with the platelet aggregation measurements, in which the platelet aggregation rate in the treatment group was significantly lower than the control group. It has been reported that CD62 expressed on the surface of activated platelet and tumor-induced platelet aggregation is involved in cancer cell growth, invasion, and metastasis.^[25]^ Therefore, our findings indicate that Shen-Cao granules might help alleviate adverse tumor activities.

This study showed that the Shen-Cao granule treatment alleviated thrombocytopenia by increasing the PLT count and the TPO level in the peripheral blood of patients after chemotherapy. In addition, Shen-Cao granules reduced platelet activation and aggregation in the tumor patients. These results provide a promising prospect for the TCM-based treatment of patients with tumors. However, the detailed mechanisms and the specific target pathways of Shen-Cao granules’ action need to be further studied.
